# Brain tissue segmentation based on MP2RAGE multi-contrast images in 7 T MRI

**DOI:** 10.1371/journal.pone.0210803

**Published:** 2019-02-28

**Authors:** Uk-Su Choi, Hirokazu Kawaguchi, Yuichiro Matsuoka, Tobias Kober, Ikuhiro Kida

**Affiliations:** 1 Center for Information and Neural Networks, National Institute of Information and Communications Technology, Osaka, Japan; 2 Graduate School of Frontier Biosciences, Osaka University, Osaka, Japan; 3 Siemens Healthcare K.K., Osaka, Japan; 4 Advanced Clinical Imaging Technology, Siemens Healthcare AG, Lausanne, Switzerland; 5 Department of Radiology, University Hospital (CHUV), Lausanne, Switzerland; 6 LTS5, École Polytechnique Fédérale de Lausanne (EPFL), Lausanne, Switzerland; University of Queensland, AUSTRALIA

## Abstract

We proposed a method for segmentation of brain tissues—gray matter, white matter, and cerebrospinal fluid—using multi-contrast images, including a T1 map and a uniform T1-weighted image, from a magnetization-prepared 2 rapid acquisition gradient echoes (MP2RAGE) sequence at 7 Tesla. The proposed method was evaluated with respect to the processing time and the similarity of the segmented masks of brain tissues with those obtained using FSL, FreeSurfer, and SPM12. The processing time of the proposed method (28 ± 0 s) was significantly shorter than those of FSL and SPM12 (444 ± 4 s and 159 ± 2 s for FSL and SPM12, respectively). In the similarity assessment, the tissue mask of the brain obtained by the proposed method showed higher consistency with those obtained using FSL than with those obtained using SPM12. The proposed method misclassified the subcortical structures and large vessels since it is based on the intensities of multi-contrast images obtained using MP2RAGE, which uses a similar segmentation approach as FSL but is not based on a template image or a parcellated brain atlas, which are used for FreeSurfer and SPM12, respectively. However, the proposed method showed good segmentation in the cerebellum and white matter in the medial part of the brain in comparison with the other methods. Thus, because the proposed method using different contrast images of MP2RAGE sequence showed the shortest processing time and similar segmentation ability as the other methods, it may be useful for both neuroimaging research and clinical diagnosis.

## Introduction

Structural information regarding brain tissue is important for both neuroimaging research and clinical diagnosis. Magnetic resonance imaging (MRI) has been widely used to obtain structural information from various types of contrast images. Different MR contrast images can show brain abnormalities via segmentation of subcortical structures in neuronal disorders, such as Parkinson’s or Alzheimer’s diseases [1]. Furthermore, gray matter (GM) segmentation can be used to estimate cortical thickness or volume to evaluate developmental stages or the effects of aging [2]. In functional MRI, white matter (WM) segmentation can provide an inflated brain mesh [3] to project brain activation maps.

Most previous MRI segmentation methods for brain tissues, including GM, WM, and cerebrospinal fluid (CSF), were based on the signal intensities in T1-weighted (T1w), T2-weighted (T2w), and proton-density (PD) images. However, these images show an intrinsic overlapping intensity distribution among brain tissues, which renders their segmentation into GM, WM, and CSF challenging. In addition, the overlap can often be substantial because of factors such as image inhomogeneity, noise, and the partial volume effect (PVE), which can reduce the precision of the segmentation [4–6]. Among these factors, PVE is a critical obstacle to effective brain tissue segmentation because although other artifacts can be improved or corrected with sophisticated pre-processing methods, such as bias field correction [7], PVE cannot be easily corrected because MR brain images consist of three dimensional cubic structures i.e., voxels, which contain multiple constituents of different brain tissues.

To overcome the influences of PVE in brain tissue segmentation, numerous groups have employed several approaches using (1) thresholding methods, (2) region-growing methods, (3) clustering, and (4) Bayesian classification [8–10]. In the thresholding method, brain tissues are defined by thresholds based on their intensity histograms. This approach is fast and efficient but may fail to accurately define brain tissues because it does not consider neighborhood information [11]. The region-growing method considers the information of neighborhood voxels by estimating their similarity to a seed voxel, which can lead to biased results because of incorrect seed point selection. The clustering methods use intensities and spatial information (i.e., neighborhood information) as features and classify brain voxels of different types of brain tissue without requiring training. A previous study increased the classification accuracy using various clustering algorithms, such as expectation–maximization or fussy C-means [5]. Finally, Bayesian classification has been adopted by several popular segmentation software packages, such as FSL, FreeSurfer, and SPM12 [12]. Zhang et al. (2001) calculated the maximum probabilities of each brain tissue and used the neighborhood voxel information by employing the Markov random field statistical model to improve brain tissue classification [13]. However, these intensity-based approaches still involve PVE MRI artifacts, particularly in the segmentation of infant brains [14] or abnormal brain structures, such as a tumor [15]. Machine-learning algorithms have also been applied to brain segmentation methods [16].

Recently, MRI systems have shifted to using ultra-high fields [UHF ≥ 7 Tesla (T)] to obtain fine-structure and functional images with sub-millimeter resolution. However, acquisition of total brain volume images at sub-millimeter resolutions increases the dataset size substantially, and the segmentation methods described above require longer computation times for such high spatial-resolution images. In addition, T1w images for brain tissue segmentations are usually obtained with a magnetization-prepared rapid gradient echo (MPRAGE) sequence, but the image may contain severe inhomogeneities at UHF. In contrast to the MPRAGE sequence, the magnetization-prepared 2 rapid acquisition gradient echoes (MP2RAGE) sequence can produce more homogeneous T1w image, termed a uniform (UNI) image, by combining two different gradient echo images with two different inversion times (TIs) [17–19]. The MP2RAGE sequence also produces images with different types of contrast, such as two gradient echo images (INV1 and INV2), with different TIs and flip angles (FAs), a T1 map, and a T1w image without a noisy background (UNIDEN).

In the present study, we proposed a segmentation method for brain tissue (GM, WM, and CSF) that used the different contrast images (INV1, INV2, UNI, and a T1 map) acquired using MP2RAGE sequences at 7 T. We evaluated the proposed method with respect to processing time and the similarity of the segmented masks of brain tissues with those from FSL, FreeSurfer, and SPM12, which are commonly used in neuroimaging protocols.

## Methods

### Subjects

Seven volunteers (four males and three females, aged 23–63 years) without a history of neurological disease or any other medical condition participated in this study after providing written informed consent. All experiments were approved by the Ethics and Safety Committees.

### MRI acquisition

The experiments were performed on a 7-T investigational MRI scanner (MAGNETOM 7T; Siemens Healthineers, Germany) with a 32-channel head coil (Nova Medical, Wilmington, MA). The MP2RAGE sequence [17] was acquired, with a work-in-progress software package from Siemens Healthineers, using the following parameters: repetition time = 5000 ms, echo time = 3.36 ms, TI1/TI2 = 800 ms/2600 ms, FA1/FA2 = 4°/5°, matrix = 320 × 320 × 256, voxel size = 0.8 × 0.8 × 0.8 mm^3^, integrated parallel acquisition techniques with parallel imaging acceleration = 3, and scan time = 8 min 23 s.

### Prerequisites: Brain extraction

The skin and skull from all MP2RAGE images were stripped to extract the brain using the BET toolbox in FSL 5.0 (FMRIB, Oxford, UK). Most images except for UNIDEN could not be automatically stripped because of their different contrasts in comparison with the conventional T1w image and the background noise in these images, such as the salt-and-pepper noise found in UNI. Therefore, the brain-extracted INV2 was used as a mask for the brain extraction process in the other images (UNI, T1, and INV1). After the INV2 mask-based brain extraction, an erosion process was used to remove residual non-brain tissues.

### FSL segmentation

Brain-extracted UNI images from all subjects were segmented using the FAST toolbox in FSL 5.0 (http://fsl.fmrib.ox.ac.uk/fsl/fslwiki/). Masks of brain tissues (GM, WM, and CSF) were constructed by binarization of probability maps, which were created in the segmentation process, with a threshold value of 0.5.

### FreeSurfer segmentation

Brain-extracted UNI images from all subjects were segmented using the auto-reconstruction processes in FreeSurfer 6.0 (https://surfer.nmr.mgh.harvard.edu/fswiki). Since skull stripping had already been performed with the BET toolbox in FSL, this step was excluded in the auto-reconstruction processes using FreeSurfer. After auto-reconstruction, we manually created masks of three brain tissues (GM, WM, and CSF) using the entire parcellated image. In addition, the brainstem, pallidum, and ventral diencephalon [20] were defined as WM for comparison with FSL and SPM12 because these areas are considered to be WM in FSL and SPM12.

### SPM12 segmentation

Brain-extracted UNI images from all subjects were segmented by a unified algorithm that included bias correction, tissue classification, and registration using SPM12 (http://www.fil.ion.ucl.ac.uk/spm/). Probability maps of brain tissues (GM, WM, and CSF) were created using medium bias regularization and a reference brain probability map; then, masks of brain tissues were constructed by binarization of the probability maps with a threshold value of 0.5.

### Evaluation of segmentation

To evaluate the performance of the segmentation, we compared the processing time and the similarity of the masks of brain tissues segmented by the proposed method with those produced with FSL, FreeSurfer, and SPM12. All computations were processed by a computer with the following specifications, and the processing times were recorded: macOS (Sierra 10.12.5), 3.5 GHz 6-Core Intel Xeon E5 CPU, and 16 GB RAM. A paired t-test was performed for comparison of processing times between the proposed method and FSL and SPM12, respectively. For the similarity evaluation, the absolute volume difference (AVD), which measures differences in voxel ratios, and dice coefficient (DICE), which measures the spatial overlap ratio of each mask of brain tissue, were calculated with Eqs 1 and 2, respectively; the values for these parameters generated by the proposed method and by the other software packages were compared.

$$AVD=\frac{\left|{Mask}_{proposed}-{Mask}_{FSL\;or\;FreeSurfer\;or\;SPM12}\right|}{{Mask}_{FSL\;or\;FreeSurfer\;or\;SPM12}}\times 100$$ (1)

$$DICE=\frac{2|{Mask}_{proposed}\cap {Mask}_{FSL\;or\;FreeSurfer\;or\;SPM12}|}{\left|{Mask}_{proposed}|+|{Mask}_{FSL\;or\;FreeSurfer\;or\;SPM12}\right|}\times 100$$ (2)

In addition, the modified Hausdorff distance (MHD), which measures boundary distances [21], was calculated using the edges of all tissue masks and compared between the proposed method and the other software packages. The MHD values for each brain tissue were calculated in the same axial slice.

A one-way ANOVA was performed for AVD, DICE, and MHD values of the three brain tissues (GM, WM, and CSF) in MATLAB R2016b (The MathWorks Inc., Natick, MA, USA). Then post-hoc tests for pairwise multiple comparisons were applied to estimate significances of the differences between evaluation measurements of different comparisons.

### Estimation of influence of noise on the proposed method

To examine the influence of noise on the proposed method, we added Gaussian noise levels of 9% to all images (S1 Fig) and performed the proposed method. The signal to noise ratio, which was evaluated with the region of interest method, in the whole GM decreased by 3.0 ± 0.3%, 20.0 ± 1.9%, and 8.4 ± 0.8% in INV1, T1, and UNI with Gaussian noise levels of 9%, respectively. The similarity of the masks of brain tissues with noise was evaluated with the masks obtained using FSL without noise as mentioned above.

## Results

### Image calculation for brain tissue segmentation

Since MP2RAGE images exhibit different contrasts for brain tissues (GM versus WM and CSF versus GM) and different scales of intensity, we normalized the intensities of the whole brain, after stripping the skin and skull, for using a feature-scaling method described by Eq 3. *S*_*raw*_, min (*S*_*raw*_), and max (*S*_*raw*_) represent the raw intensities, the minimum intensity, and the maximum intensity in each MP2RAGE image, respectively.

$$S_{normalized}=\left(\frac{S_{raw}-min(S_{raw})}{\max\left(S_{raw}\right)-min(S_{raw})}\right)$$ (3)

**Fig 1:**
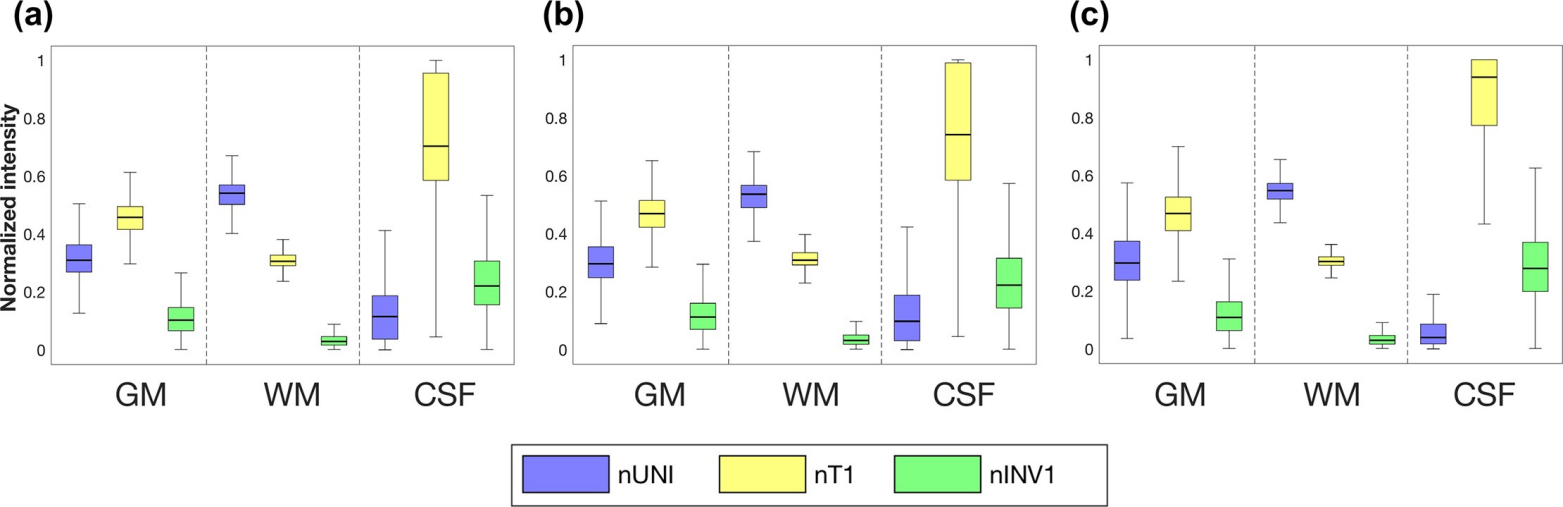
Intensities in brain tissues. Box plots show the intensities of nUNI, nT1, and nINV1 extracted from the tissue masks using (a) FSL, (b) FreeSurfer, and (c) SPM12.

**Fig 2:**
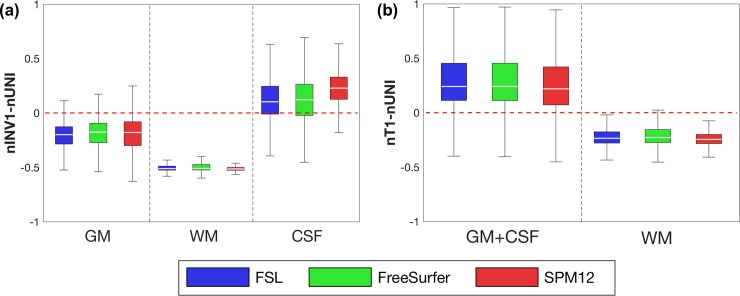
Calculated intensities in brain tissues. Box plots show (a) (nINV1 − nUNI) values and (b) (nT1 − nUNI) values from the FSL, FreeSurfer, and SPM12 masks.

We found that each brain tissue produced different normalized intensities based on the different MP2RAGE images, normalized T1 (nT1), normalized INV1 (nINV1), and normalized UNI (nUNI). To evaluate the relationship of the intensities between different MP2RAGE images, the intensities of nUNI, nT1, and nINV1 were extracted from all masks of brain tissue segmented using FSL, FreeSurfer, and SPM12 (Fig 1). CSF exhibited larger intensity distributions in the order nT1 > nINV1 > nUNI. GM exhibited larger intensity distributions in the order nT1 > nUNI > nINV1. WM showed larger intensity distributions in the order nUNI > nT1 > nINV1. The calculated values of (nINV1 − nUNI) and (nT1 − nUNI) can clarify the relationships of the normalized intensities between the brain tissues (Fig 2).

**Fig 3:**
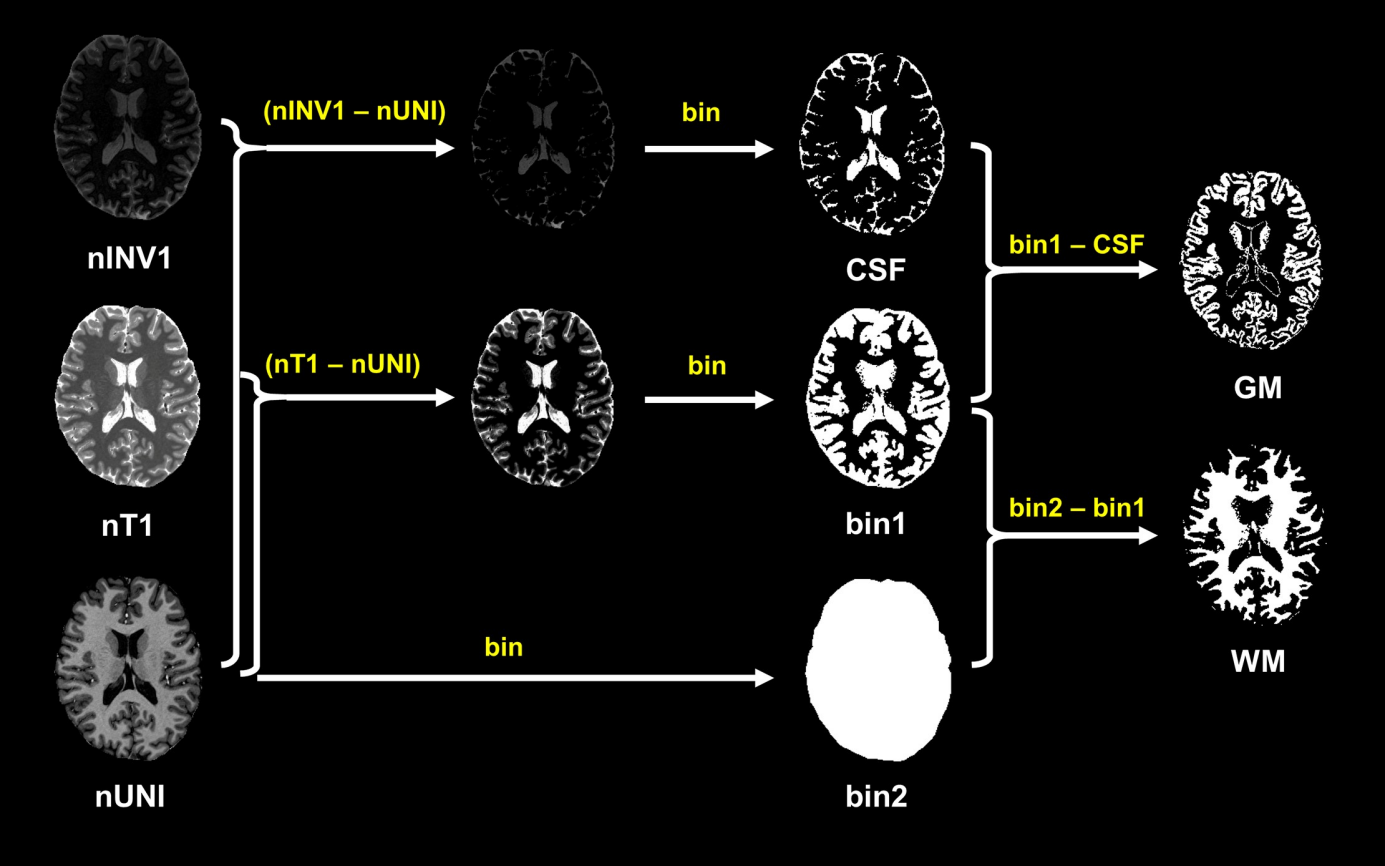
Scheme of the proposed method. nT1: normalized T1, nINV1: normalized INV1, nUNI: normalized UNI, bin: binarization.

Based on the different contrast relationships between simultaneously acquired images, we proposed the segmentation of the brain tissues. Each mask of brain tissue was calculated using Eqs 4–6. Binarization with a threshold of 0 is represented by “bin.” A scheme of the proposed method is shown in Fig 3.

$$CSF=bin\;(n{INV1}_{extracted}-{nUNI}_{extracted})$$ (4)

$$GM=bin\;({nT1}_{extracted}-{nUNI}_{extracted})-CSF$$ (5)

$$WM=bin\;({nUNI}_{extracted})-(CSF+GM)$$ (6)

### Processing time

**Fig 4:**
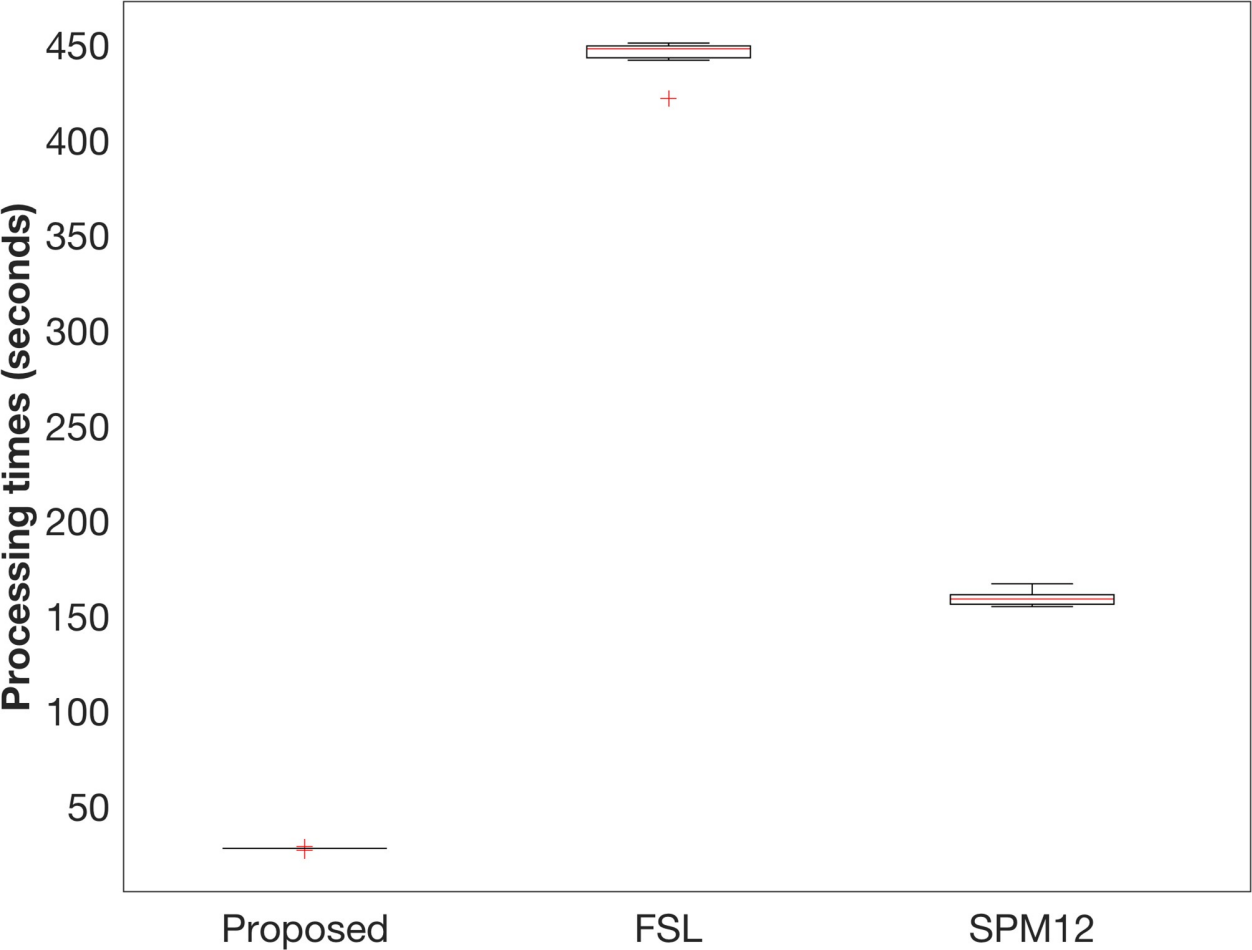
Processing times for the proposed method, FSL, and SPM12. Box plots show the processing times for all subjects with the proposed method, FSL, and SPM12. The red cross indicates outliers.

The average processing times for the segmentation were 28 ± 0 s with the proposed method, 444 ± 4 s with FSL, and 159 ± 2 s with SPM12 (Fig 4). The processing time of the proposed method was significantly shorter than that of the other packages (p < 0.001 vs. FSL and p < 0.001 vs. SPM12, paired *t*-test). The processing time with FreeSurfer was excluded because it included several processing steps beyond brain tissue segmentation, such as the reconstruction of the inflated brain, segmentation of the subcortical structure, and calculation of cortical thickness.

### Similarity evaluation

**Fig 5:**
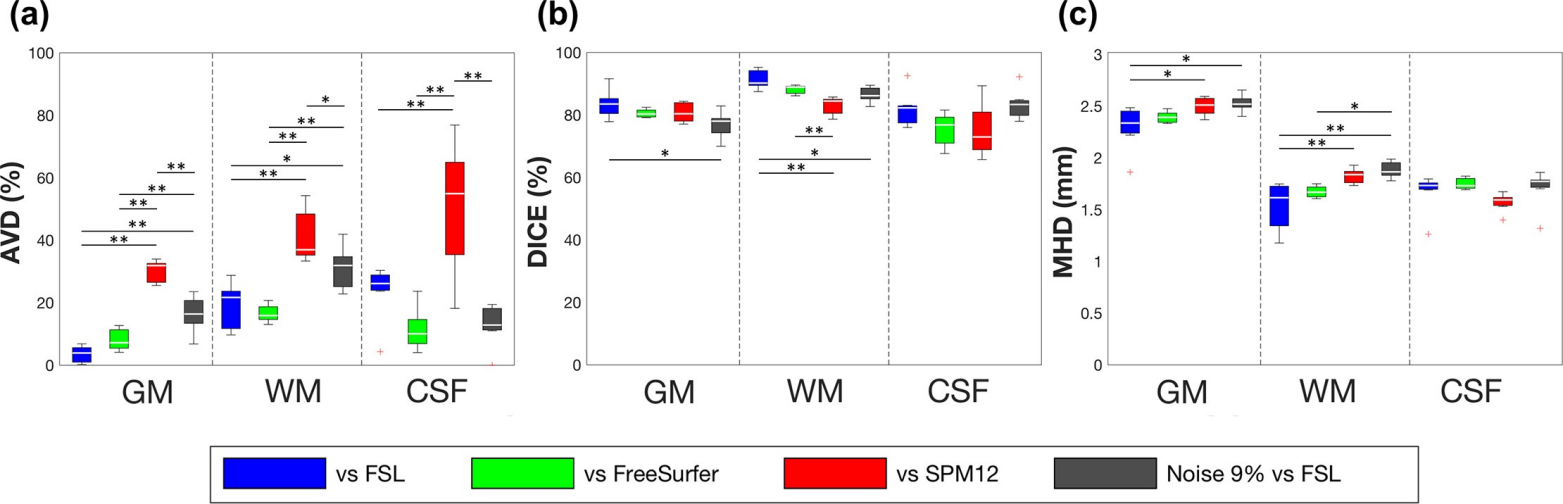
Similarity of segmentation with FSL, FreeSurfer, and SPM12. Box plots show (a) AVD, (b) DICE, and (c) MHD between the proposed method and the other methods. In addition, the segmentation at a noise level of 9% in images with the proposed method was evaluated with that using FSL at no noise. The red cross indicates outliers. AVD: absolute volume difference, DICE: dice coefficient, MHD: modified Hausdorff distance. *p < 0.05, **p < 0.01.

**Fig 6:**
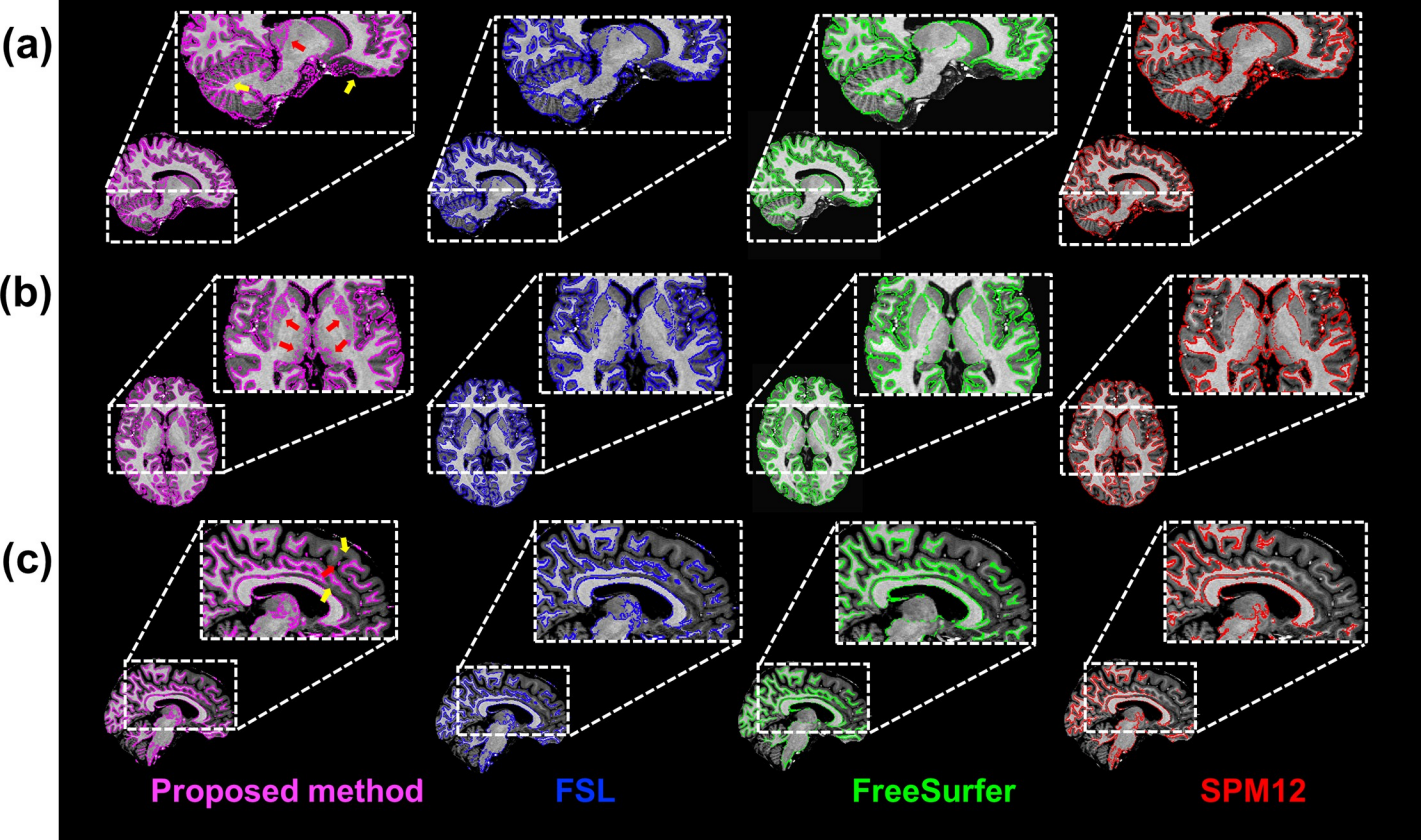
Segmentation performance using the proposed method, FSL, FreeSurfer, and SPM12. (a) Gray matter (GM) segmentations in the medial part of the brain and (b) subcortical regions. (c) White matter (WM) segmentation in the medial part of the brain. The yellow and red arrows indicate superior or inferior segmentation of GM and WM with the proposed method compared with the other methods, respectively.

Fig 5 shows the evaluation of the proposed method in comparison with FSL, FreeSurfer, and SPM12 with respect to the AVD, DICE, and MHD of the segmented masks of whole brain tissues. The spatial differences, i.e., AVD, between the proposed method and FreeSurfer were <20% in all three brain tissues, but the differences were significantly larger (30%–50%) in comparison with SPM12 (Fig 5A). The GM masks segmented using the proposed method almost completely corresponded to those produced using FSL, indicating that AVD was 3.5% ± 1.0%. The average DICEs for GM and WM segmentation between the proposed method and all other methods were >80%, whereas those for CSF segmentation were approximately 75%–82% (Fig 5B). The average DICEs for all brain tissues between the proposed method and FSL were slightly higher than those with FreeSurfer and SPM12. The average MHDs for GM, i.e., 2.3–2.5 mm, between the proposed method and all other methods were larger than those for WM and CSF, i.e., 1.5–1.8 mm (Fig 5C). The similarities between the proposed method and the other packages were supported by the comparison with the manual segmentation, which was performed on a single slice image in all subjects (S2 Fig). The similarities in GM and WM in the proposed method with manual segmentation were similar to those with FSL, FreeSurfer but not SPM12 (S3 Fig). As for the influence of noise levels, the similarities in AVD, DICE, and MHD in comparison with FSL linearly deteriorated with increasing noise levels in all contrast images of MP2RAGE. However, at a noise level of 9%, the similarities in AVD and DICE were still better than those with SPM12 (Fig 5). Larger differences in segmentation in comparison with SPM12 were found in the GM in the inferior frontal gyrus, cerebellum (Fig 6A), subcortical structures (Fig 6B), large vessels (Fig 6B and 6C), and WM in the medial part of the brain areas (Fig 6C). GM in the inferior frontal gyrus and cerebellum, and WM in the medial part of the brain areas were well segmented with the proposed method, whereas subcortical structures and large vessels were misclassified.

## Discussion

We proposed a segmentation method with a significantly shorter processing time for brain tissues (GM, WM, and CSF). The proposed method employs a simple calculation of normalized signal intensities in the images with differing contrasts produced with an MP2RAGE sequence (UNI, INV1, and T1). The calculations were based on a consistent and specific tissue-dependent pattern of normalized intensities in the masks segmented with FSL, FreeSurfer, and SPM12, which are commonly used in neuroimaging protocols. Most segmentation methods use a single T1w image obtained from an MPRAGE sequence, and numerous groups have attempted to solve the problem of overlapping signal distribution with complex and sophisticated methods that require considerable processing time to classify different brain tissues [5,13]. Although a few segmentation methods using multiple contrast images from an MP2RAGE sequence have been proposed [22], these require longer processing times because of the complexity of their algorithms. In contrast, the proposed method exhibits superior processing times because it utilizes simple calculations and three different contrast images from an MP2RAGE sequence. Therefore, the proposed method can segment a mask of brain tissues from the images, even with a high spatial-resolution at UHF, with shorter processing times.

To evaluate the segmentation performance of the proposed method, we calculated the AVD, DICE, and MHD values and compared them with other methods: FSL, FreeSurfer, and SPM12. We found differences between the proposed method and the other methods, but the brain tissue segmentation in the proposed method resembled that using FSL than those using FreeSurfer and SPM12. These differences in comparison with the other methods could be due to the differences in segmentation approaches in each method. Segmentation using the proposed method and FSL is based on the image intensities, whereas FreeSurfer and SPM12 use a template image as a reference for brain tissue and a parcellated brain atlas, respectively. The atlas-based segmentation approach can define subcortical structures, such as the putamen and caudate, and classify large blood vessels, resulting in its dissimilarity to the proposed method. In contrast, the GM and WM in the cerebellum and WM in the medial part of the brain areas showed good segmentation with the proposed method in comparison with the other methods, which also resulted in some of the comparative dissimilarities. Overall, the dissimilarities with the other methods do not imply that the proposed method is inferior or superior to the other methods because there is no gold standard software package for brain segmentation. However, it is important to note that a comparison with the manual method indicates that the segmentation of the proposed method is comparable to the other methods.

Our approach had some limitations as well as some potential for further development. One limitation was the poor segmentation of subcortical structures and large vessels. Because we employ different contrast images from an MP2RAGE sequence for segmentation and simple calculations, the performance of the proposed method depends on the relationship of the normalized intensities of the images and compensation values (i.e., thresholding) in the calculation of segmentation. MP2RAGE sequence can produce more homogeneous T1w image and T1 map, however, B1 inhomogeneities at UHF could not be completely canceled by combining two different gradient echo images with two different inversion times, INV1 and INV2. The spatial distribution of B1 inhomogeneities at UHF shows a central bright spot and lower B1 intensity around the spot, which correspond to the subcortical regions and cerebral cortex in the human brain, respectively. B1 correction of MP2RAGE images using a post-process could be used for improvement of the contrast in the subcortical regions [23]. However, since post-hoc B1 correction decreases T1 values and increases the signal intensity of T1w images in most brain regions, the relationship of the normalized intensities of these images can be changed for poor segmentation in the present acquisition parameters (S4 Fig). Therefore, the segmentation of the subcortical structures could be improved using different relationships of normalized intensities with optimized acquisition parameters, e.g., TIs and FAs for post-hoc B1 correction [18, 24, 25] and using optimized compensation values in the segmentation calculation. Despite these limitations, the proposed method has the potential for use in segmentation because of the good segmentation of the cerebellum and WM in the medial part of the brain and the significantly reduced processing time. Although the boundary between GM and WM in the cerebellum is narrow and complex, due to the small size of its cytoarchitectural features [26], the cerebellum could be well segmented with the proposed method. Accurate segmentation of the cerebellum is useful for the reconstruction of an inflated image and the diagnosis of cerebellar WM diseases. The substantially shortened processing time is a critical factor because several studies have attempted to reduce the processing time of segmentation for clinical diagnosis while maintaining acceptable accuracy [27] or allowing precise segmentation [28]. The proposed method can act as a quick reference for further, more precise brain segmentation approaches. The advantage of the shortened processing time will be more evident when using large datasets, such as those generated with ultra-high spatial resolution. In addition, although previous studies have suggested using both T1w and other MR images, such as PD, T2w, or fluid-attenuated inversion recovery images [9,29,30], for segmentation, these multi-spectrum segmentation approaches run the risk of head motion between scans. Because the proposed method uses different contrast images obtained simultaneously using an MP2RAGE sequence, it is free from the effects of possible head motion.

## Conclusions

We propose a novel brain tissue segmentation method that uses different contrast images from an MP2RAGE sequence. The proposed method allows rapid processing with relevant segmentation of brain tissues. A recent study has reported MP2RAGE-based segmentation using two images with different inversion times, i.e., INV1 and INV2 [31]. The study demonstrated superior segmentation in subcortical structures in comparison with FSL and SPM12, whereas our proposed method produced good segmentation in the cerebellum and WM in the medial part of the brain. Taken together, MP2RAGE-based segmentation is highly dependent on the relationship of the different contrasts between the input images used in the calculations, and this property could be of benefit in acquiring more precise focal segmentation of specific brain structures, such as the subcortical structures and the cerebellum. Thus, the MP2RAGE-based segmentation we proposed here has the potential to be applied, with optimized parameters of the MP2RAGE sequence, to both neuroimaging research and clinical diagnosis.

## Supporting information

S1 Fig Examples of MP2RAGE images without and with 9% noise level and the segmentation image. (a) First inversion gradient echo image (INV1), (b) T1-weighted image (UNI), (c) T1 map (T1), (d) segmentation image with the proposed method.
(TIF)

S2 Fig Segmentation of three tissues with the manual method, the proposed method, FSL, FreeSurfer, and SPM12. (TIF)

S3 Fig Similarity of segmentation with the manual method. Box plots show (a) AVD, (b) DICE, and (c) MHD between the manual method and the other methods, including the proposed method. The red cross indicates outliers. AVD: absolute volume difference, DICE: dice coefficient, MHD: modified Hausdorff distance. *p < 0.05, **p < 0.01.
(TIF)

S4 Fig Calculated intensities of the brain tissues. Box plots show (a) (nINV1 − nUNI) values and (b) (nT1 − nUNI) values from the FSL segmented masks.
(TIF)
