# Supplementary figures and images for: Brain tissue segmentation based on MP2RAGE multi-contrast images in 7 T MRI
Source: PLoS One. 2019 Feb 28;14(2):e0210803. doi: 10.1371/journal.pone.0210803 (PMC6394968; doi:10.1371/journal.pone.0210803)

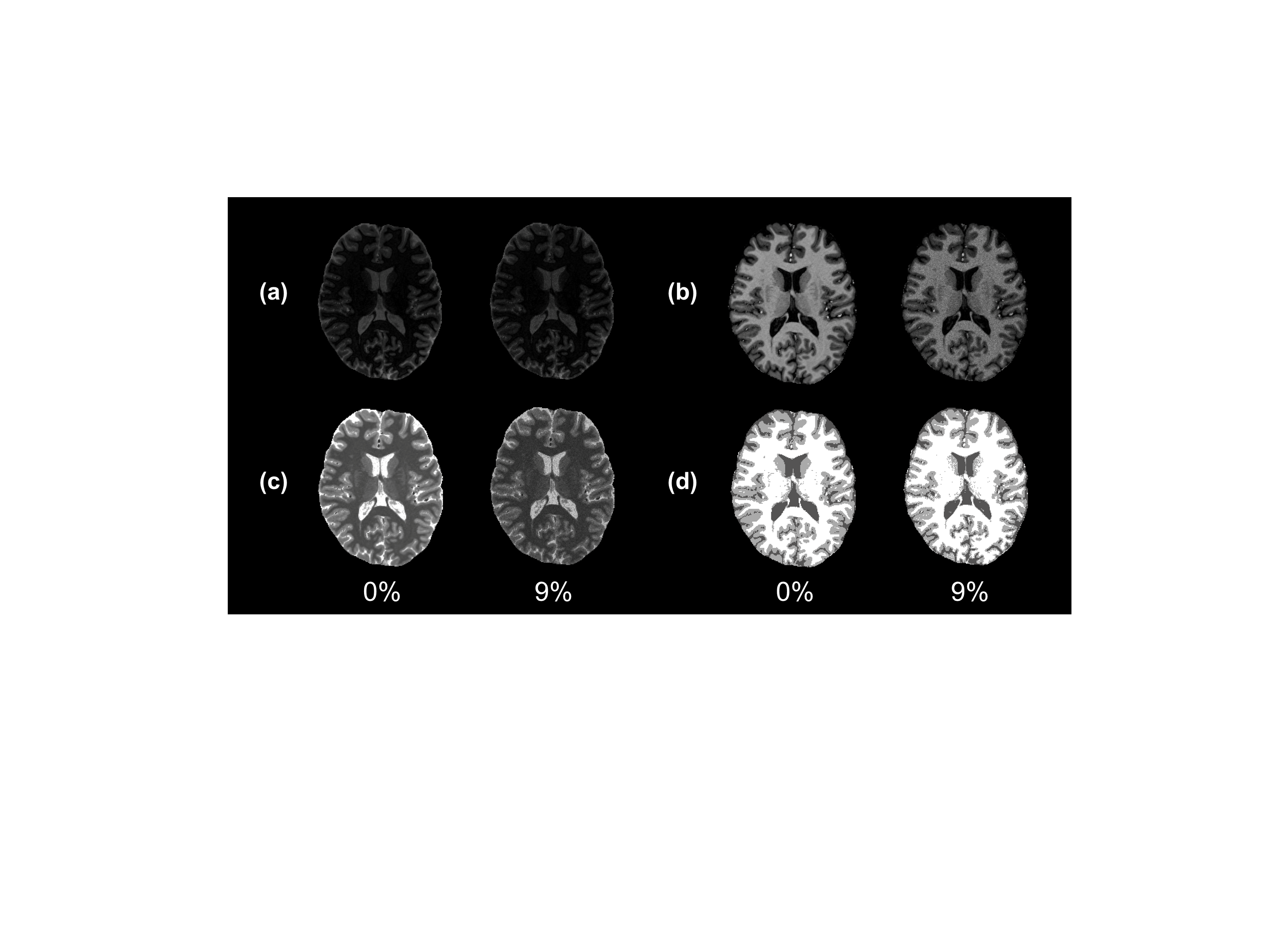

Supplement: S1 Fig — (a) First inversion gradient echo image (INV1), (b) T1-weighted image (UNI), (c) T1 map (T1), (d) segmentation image with the proposed method. (TIF) [file pone.0210803.s001.TIF]

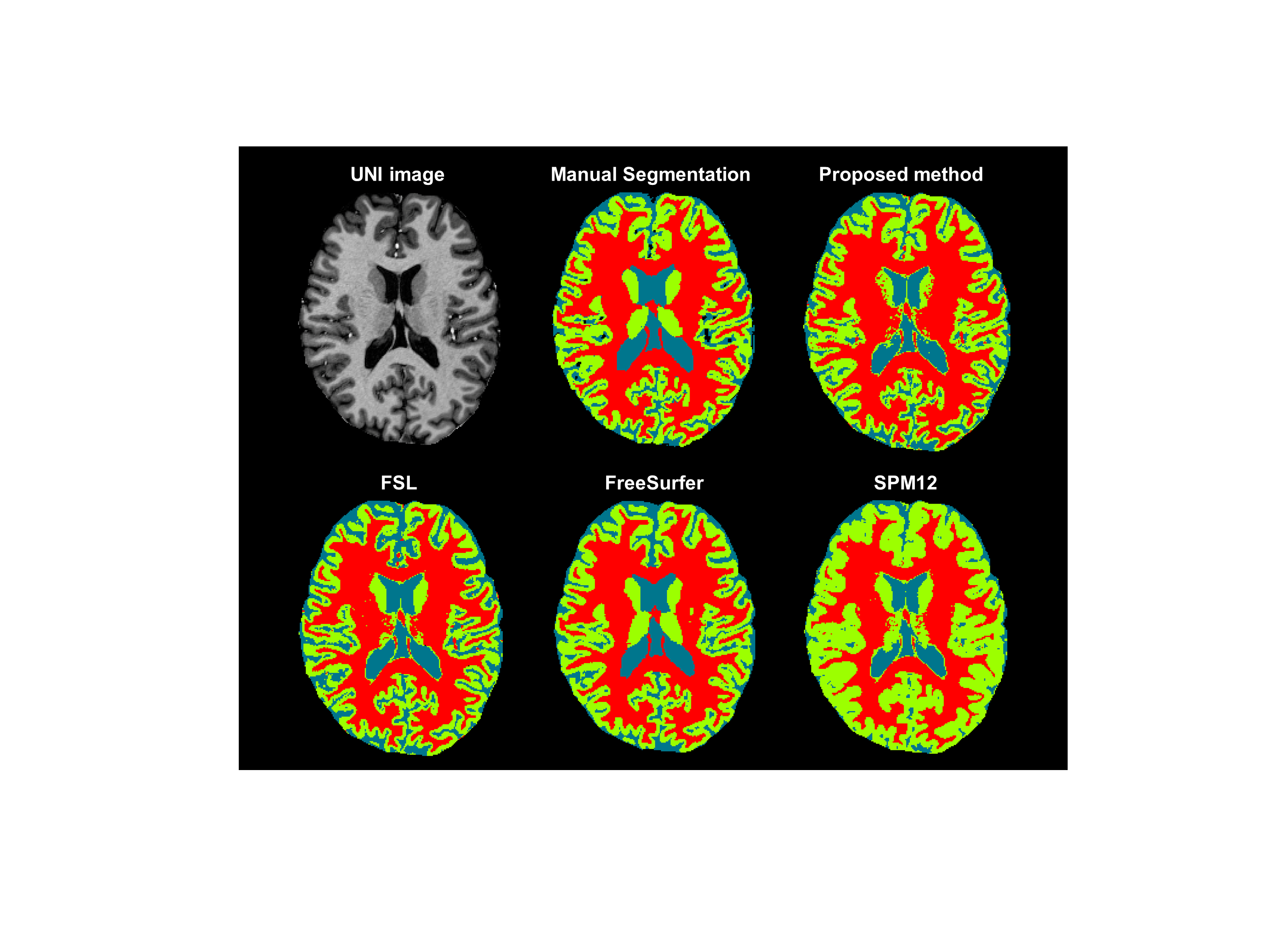

Supplement: S2 Fig — (TIF) [file pone.0210803.s002.TIF]

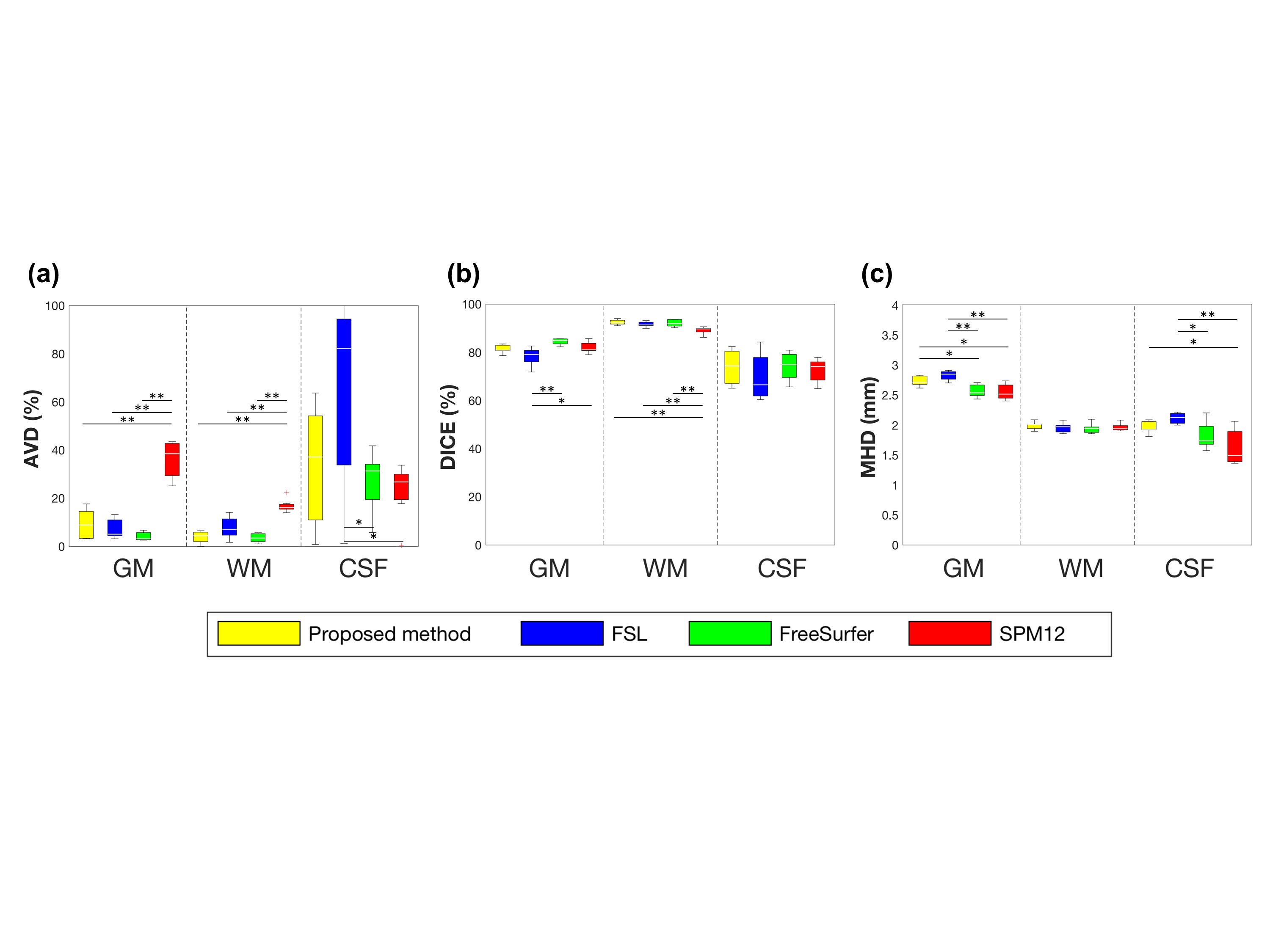

Supplement: S3 Fig — Box plots show (a) AVD, (b) DICE, and (c) MHD between the manual method and the other methods, including the proposed method. The red cross indicates outliers. AVD: absolute volume difference, DICE: dice coefficient, MHD: modified Hausdorff distance. *p < 0.05, **p < 0.01. (TIF) [file pone.0210803.s003.TIF]

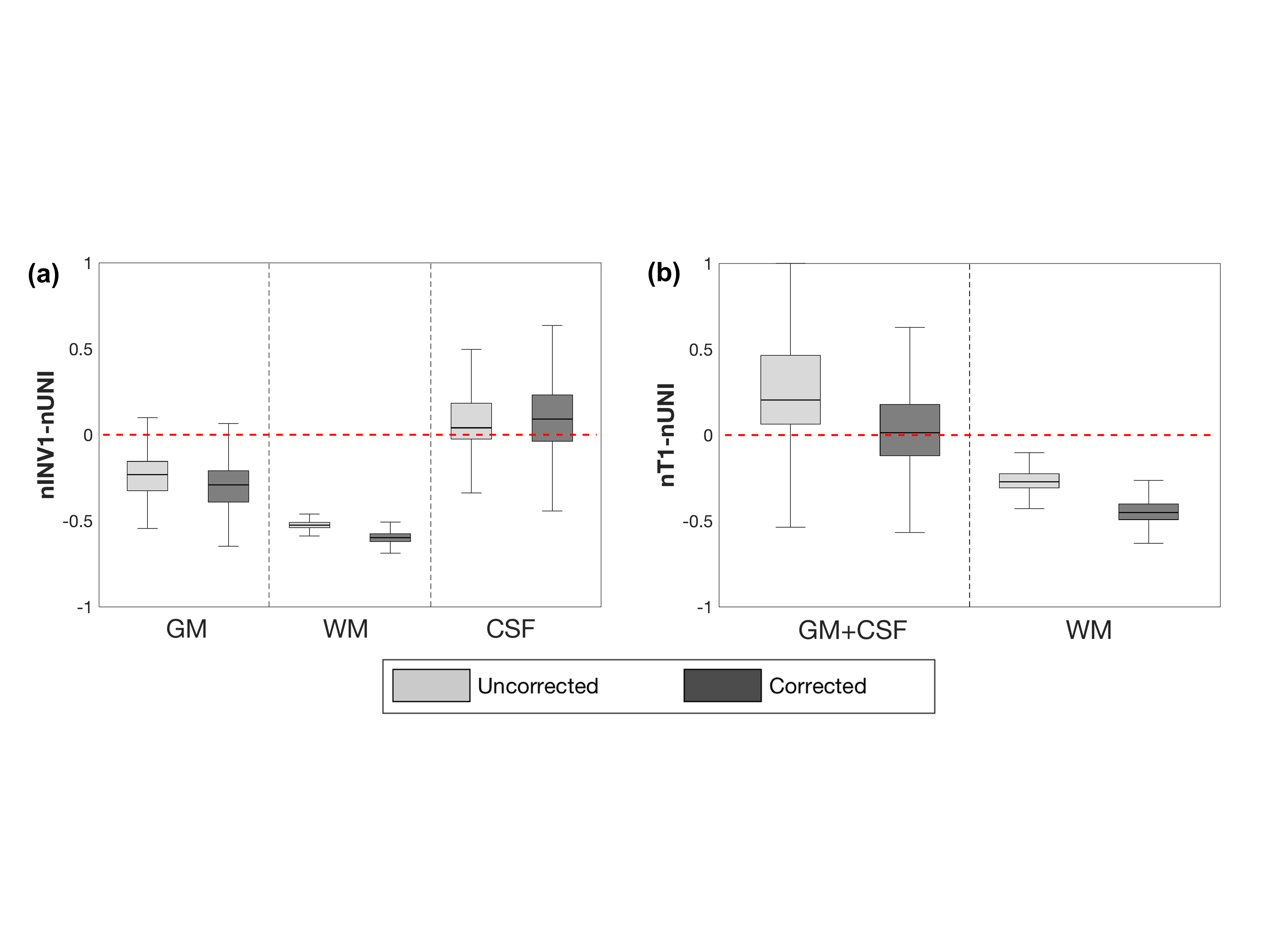

Supplement: S4 Fig — Box plots show (a) (nINV1 − nUNI) values and (b) (nT1 − nUNI) values from the FSL segmented masks. (TIF) [file pone.0210803.s004.TIF]
